# Pneumococcal carriage and antibiotic susceptibility patterns from two cross-sectional colonization surveys among children aged <5 years prior to the introduction of 10-valent pneumococcal conjugate vaccine — Kenya, 2009–2010

**DOI:** 10.1186/s12879-016-2103-0

**Published:** 2017-01-05

**Authors:** Miwako Kobayashi, Laura M. Conklin, Godfrey Bigogo, Geofrey Jagero, Lee Hampton, Katherine E. Fleming-Dutra, Muthoni Junghae, Maria da Gloria Carvalho, Fabiana Pimenta, Bernard Beall, Thomas Taylor, Kayla F. Laserson, John Vulule, Chris Van Beneden, Lindsay Kim, Daniel R. Feikin, Cynthia G. Whitney, Robert F. Breiman

**Affiliations:** 1 Epidemic Intelligence Service, Centers for Disease Control and Prevention, Atlanta, GA USA; 2 Division of Bacterial Diseases, Centers for Diseases Control and Prevention, 1600 Clifton Road NE, MS C-25, Atlanta, GA 30329-4027 USA; 3 Centre for Global Health Research, Kenya Medical Research Institute, Kisumu, Kenya; 4 International Emerging Infections Program, Centers for Disease Control and Prevention, Nairobi, Kenya; 5 Center for Global Health, Centers for Disease Control and Prevention, Atlanta, GA USA; 6 Emory Global Health Institute, Emory University, Atlanta, GA USA

**Keywords:** *Streptococcus pneumoniae*, 10-valent pneumococcal conjugate vaccine, Colonization, Kenya, Antibiotic nonsusceptibility

## Abstract

**Background:**

Pneumococci are spread by persons with nasopharyngeal colonization, a necessary precursor to invasive disease. Pneumococcal conjugate vaccines can prevent colonization with vaccine serotype strains. In 2011, Kenya became one of the first African countries to introduce the 10-valent pneumococcal conjugate vaccine (PCV10) into its national immunization program. Serial cross-sectional colonization surveys were conducted to assess baseline pneumococcal colonization, antibiotic resistance patterns, and factors associated with resistance.

**Methods:**

Annual surveys were conducted in one urban and one rural site during 2009 and 2010 among children aged <5 years. To reflect differences in vaccine target population, recruitment was age-stratified in Kibera, whereas a simple random sample of children was drawn in Lwak. Nasopharyngeal swabs were collected from eligible children. Pneumococci were isolated and serotyped. Antibiotic susceptibility testing was performed using the 2009 isolates. Antibiotic nonsusceptibility was defined as intermediate susceptibility or resistance to ≥1 antibiotics (i.e., penicillin, chloramphenicol, levofloxacin, erythromycin, tetracycline, cotrimoxazole, and clindamycin); multidrug resistance (MDR) was defined as nonsusceptibility to ≥3 antibiotics. Weighted analysis was conducted when appropriate. Modified Poisson regression was used to calculate factors associated with antibiotic nonsusceptibility.

**Results:**

Of 1,087 enrolled (Kibera: 740, Lwak: 347), 90.0% of these were colonized with pneumococci, and 37.3% were colonized with PCV10 serotypes. There were no differences by survey site or year. Of 657 (of 730; 90%) isolates tested for antibiotic susceptibility, nonsusceptibility to cotrimoxazole and penicillin was found in 98.6 and 81.9% of isolates, respectively. MDR was found in 15.9% of isolates and most often involved nonsusceptibility to cotrimoxazole and penicillin; 40.4% of MDR isolates were PCV10 serotypes. In the multivariable model, PCV10 serotypes were independently associated with penicillin nonsusceptibility (Prevalence Ratio: 1.2, 95% CI 1.1–1.3), but not with MDR.

**Conclusions:**

Before PCV10 introduction, nearly all Kenyan children aged <5 years were colonized with pneumococci, and PCV10 serotype colonization was common. PCV10 serotypes were associated with penicillin nonsusceptibility. Given that colonization with PCV10 serotypes is associated with greater risk for invasive disease than colonization with other serotypes, successful PCV10 introduction in Kenya is likely to have a substantial impact in reducing vaccine-type pneumococcal disease and drug-resistant pneumococcal infection.

**Electronic supplementary material:**

The online version of this article (doi:10.1186/s12879-016-2103-0) contains supplementary material, which is available to authorized users.

## Background

*Streptococcus pneumoniae* (pneumococcus) is a leading cause of morbidity and mortality worldwide [1–3]. The World Health Organization estimates that pneumococcal infection is a cause of 476,000 deaths of HIV negative children aged <5 years every year globally [4]. Low income countries have both the highest rate of pneumococcal mortality and the largest number of total deaths from pneumococcal disease among children [4, 5].

Pneumococci colonize the upper respiratory tract, a necessary precursor to pneumococcal pneumonia and invasive pneumococcal disease (IPD) [6]. Pneumococcal colonization first occurs early in life, and colonization rates peak among children of preschool age [7, 8]. Transmission of colonization is common from person to person through respiratory secretions, particularly within families and other groups in which people are in close contact with each other [9–12]. Groups at highest risk for IPD include young children, the elderly, and immunocompromised individuals, such as those infected with HIV [13].

The effectiveness of pneumococcal conjugate vaccines (PCVs) in reducing the incidence of IPD has been well documented [14–20]. Of more than 90 pneumococcal serotypes, a limited number of serotypes cause most IPD in children [21, 22]; PCVs include the serotypes most commonly causing invasive disease. PCVs not only reduce nasopharyngeal carriage of vaccine-type pneumococci among the vaccinated, but these vaccines also produce a herd effect by interrupting subsequent transmission to unvaccinated individuals [23–25]. However, the cost of PCVs is a barrier to their introduction in some countries [26]. In January 2011, Kenya introduced the 10-valent pneumococcal conjugate vaccine (PCV10) into its childhood national immunization program [27, 28], with financial support provided by the Gavi Alliance. Since Kenya was one of the first African countries to introduce a PCV, whether the substantial reductions in IPD and herd effects seen in the U.S. and European countries would also be seen in Kenya was unknown, given differences in pneumococcal colonization prevalence, factors that favor pneumococcal transmission (e.g. crowding, poverty), serotype distribution [1, 29–31], higher prevalence of underlying medical conditions (e.g., HIV, malnutrition etc.) [32], and the larger proportion of young children in Kenya’s demographic profile [1].

Before PCV10 introduction, we conducted two serial cross-sectional surveys of pneumococcal colonization among children aged <5 years in two sites in Kenya, one urban and one rural. The objectives of these surveys were to establish the baseline proportion of children aged <5 years who were colonized with pneumococci, particularly pneumococci whose serotypes were included in PCV10, to describe any differences in the colonization prevalence by survey site and year, to describe antibiotic susceptibility patterns of colonizing pneumococci prior to PCV10 introduction, and to assess factors associated with antibiotic nonsusceptibility.

## Methods

### Study setting and survey population

The Kenya International Emerging Infections Program (IEIP) has been conducting Population-Based Infectious Disease Surveillance (PBIDS) since late 2005 in two geographically distinct regions: Kibera and Lwak [33]. The surveillance system was established through a collaboration between the Kenya Medical Research Institute (KEMRI) and the U.S. Centers for Disease Control and Prevention (CDC) [33, 34]. Kibera is a densely-populated urban settlement within Kenya’s capital, Nairobi. Approximately 26,000 people live in this site, and about 3,500 are children aged <5 years [33]. Most employed residents are casual laborers, servants, or small-business merchants within the city [35]. Malaria is not endemic due to the elevation, and HIV prevalence among adults is estimated to be 15% [33]. Lwak is located in rural western Kenya, and its population (population size about 29,000, 4,200 of whom are children aged <5 years [33]), is widely dispersed and predominantly consists of subsistence farmers and fishermen [35]. Malaria is endemic in the area, and the HIV prevalence among adults is estimated to be 18.5% [33].

### Cross-sectional survey

During October and November of 2009 and 2010, we performed two cross-sectional surveys among children aged <5 years in Kibera and Lwak. The first cross-sectional survey was completed in 2009 to gather baseline information before the planned vaccine introduction in 2010. Because the vaccine introduction was delayed to 2011, a smaller colonization survey was completed in 2010 to identify any potential natural shift of vaccine-type colonization between the 2 years. Study participants were selected and enrolled from the PBIDS population. When PCV10 was introduced in 2011, children aged <1 year were the target population for both Kibera and Lwak for routine vaccination. In Lwak, a ‘catch-up’ campaign was implemented that targeted 1–4 year-old children, therefore, all children aged <5 years were vaccine targets. Because of the differences in the vaccine target population, sampling strategies were different by site: recruitment was age-stratified in Kibera, and computerized IEIP records were used to draw a random sample of children aged <1 year and children ages 1–4 years. In Lwak, a simple random sample of all children aged <5 years was drawn using Lwak IEIP computerized records.

We excluded children who had not resided in the community for at least 4 months prior to the survey, had naso-facial deformities which precluded collection of a nasopharyngeal (NP) swab, or were determined to have a current illness requiring hospital admission.

In both sites, community health workers visited the homes of all selected children and invited the children to participate in the study. Caregivers were given an appointment card for enrollment of the selected child at a designated fieldwork site (Tabitha Clinic for Kibera and Lwak Mission Hospital for Lwak). Children were not replaced if they did not appear on the scheduled day. No incentives were provided to the participants; however, reimbursement for transportation costs to the clinic (Lwak) or an equivalent amount in food or a food voucher (Kibera) were given to all invited participants regardless of whether they chose to participate or not. Written informed consent was obtained either from the parent or the guardian of all participating children prior to enrollment. Upon enrollment, caregivers were asked questions on household characteristics (e.g., household size and number of children age <5 at home), smoke exposure (e.g., tobacco, lighting, and heating), cooking practices, and antibiotic usage and respiratory illness within 30 days of the survey (Additional files 1 and 2).

### Laboratory methods

NP specimens were collected from the participant’s posterior nasopharynx by trained study nurses using calcium alginate swabs, as previously described [36]. NP swabs were immediately placed in 1.0 ml skim milk-tryptone-glucose-glycerol transport medium and placed in a cool box as per the World Health Organization’s consensus methods [37]. Within 6 h, specimens were vortexed to disperse the organisms from the swab and stored at -70 °C at each study site in preparation for transport to the KEMRI laboratory.

Pneumococcal isolation was conducted at the KEMRI laboratory in Kisumu, Kenya. Supplemented Todd-Hewitt broth (STHB) containing 0.5% yeast extract combined with 1.0 mL of rabbit serum was used for the broth enrichment step to enhance pneumococcal growth [38]. Optochin susceptibility and bile solubility testing were conducted on any alpha-hemolytic colony potentially identifiable as *S. pneumoniae* [39]. In cases where more than one potential pneumococcal colony type was identified per plate, representatives of each colony morphology were selected for further testing.

For pneumococcal isolates collected from the 2009 survey, pneumococcal isolates were batched and transported on dry ice to the CDC laboratory in Atlanta, Georgia for serotyping. Serotyping was conducted using latex agglutination and the Quellung reaction. For isolates collected in 2010, multiplex PCR-based serotype was conducted at KEMRI laboratory in Kisumu followed by quality control testing by Quellung reaction performed at CDC-Atlanta laboratory for all vaccine-type isolates, all PCR non-resolved serogroups (e.g., 6A/6B/6C/6D, 7C/7B), all PCR non-typeables, and 10% of all PCR resolved serotypes. Antibiotic susceptibility testing was completed during the 2009 survey only. Antibiotic susceptibility testing for commonly used antibiotics (i.e., penicillin, chloramphenicol, levofloxacin, erythromycin, ceftriaxone, tetracycline, cotrimoxazole, and clindamycin) was performed at KEMRI or CDC-Atlanta laboratories by broth microdilution (Trek Diagnostics, Cleveland OH) according to the manufacturer’s instructions.

### Definitions

Pneumococcal serotypes were classified according to whether the serotypes were contained in either PCV10 (serotypes 1, 4, 5, 6B, 7 F, 9 V, 14, 18C, 19 F, 23 F) or the 13-valent PCV (PCV13; serotypes in PCV10 plus serotypes 3, 6A, and 19A). When multiple pneumococcal serotypes were identified from a specimen, participants were classified as colonized by a vaccine serotype if at least one serotype was contained in the specific vaccine.

Antibiotic susceptibility was determined using 2012 Clinical and Laboratory Standards Institute (CLSI) criteria for minimum inhibitory concentrations (MIC) [40]. Criteria for oral penicillin were used for penicillin (susceptible: ≤0.06 μg/ml, intermediate: 0.12–1 μg/ml, resistant: ≥2 μg/ml). Intermediate and resistant isolates were designated as nonsusceptible. An isolate was considered multidrug resistant, or MDR, if it was nonsusceptible to three or more of the following antibiotics: penicillin or ceftriaxone, chloramphenicol, levofloxacin, erythromycin, tetracycline, cotrimoxazole, and clindamycin.

### Data management and analysis

Sample size was based on the ability to measure a change in colonization of vaccine (PCV10) serotypes between the baseline colonization survey reported here and planned later surveys. We estimated that among children who are targeted to receive PCV10 (i.e., children age <1 year in Kibera and age <5 years in Lwak), a sample size of 113 children age <1 year for Kibera and 182 children age <5 years for Lwak would allow 80% power to detect a 45% reduction in vaccine-type carriage. Among children who are not targeted to receive vaccine (i.e., children age 1–4 years in Kibera), 447 children ages 1–4 years in Kibera would allow 80% power to detect a 30% reduction in vaccine-type carriage.

Descriptive analyses of participants and serotype distributions were completed, and results were compared by site. Weighted analyses were used to account for sampling differences by site when applicable, where the weighting variable was calculated as the inverse to the participants’ probability of being enrolled in the survey. The probability of non-response among those who were recruited by the community health workers was not available and thus no﻿t accounted fo﻿r, although in general, participation rate was high. Rao-Scott Chi-Square test was used for categorical variables with cell counts of ≥5, and the cell means model was used for continuous variables. We calculated prevalence ratios (PR) with 95% confidence intervals (CI) to assess factors associated with colonization with antibiotic nonsusceptible pneumococci. Because the outcomes of interest were non-rare events, Poisson regression models with modifications as described by Behrens et al. were used to estimate the PR of various risk factors associated with these outcomes [41, 42]. Variables included in the models were selected based on risk factors previously described in the literature [43–45]. Analyses were performed using SAS software (version 9.3; SAS Institute, Cary, NC).

### Ethical considerations

The study was approved by ethics committees at KEMRI and CDC.

## Results

A total of 1,087 children (Kibera 740 [514 in 2009 and 226 in 2010], and Lwak 347 [183 in 2009 and 164 in 2010]) were enrolled during the two surveys in 2009 and 2010. Kibera children were younger, had more people sleeping in the same room as the child, and lived in the community for a shorter duration (all *P* <0.0001; Table 1). In addition, Kibera children were less likely to attend school or daycare compared to Lwak children (*P* <0.0001).

**Table 1:** Characteristics of surveyed children in Kibera and Lwak, 2009–2010 surveys combined

Characteristic	Total (*N* = 1,087)	Kibera (*N* = 740)	Lwak (*N* = 347)	*P* value, Kibera vs. Lwak
Female gender, n (Weighted %; 95% CI)	547 (49.8; 46.5–53.1)	368 (48.2; 44.2–52.2)	179 (51.5; 43.2–55.8)	0.33
Weighted mean age in months (95% CI)	30.8 (29.9–31.8)	28.6 (27.5–29.6)	33.4 (31.8–35.0)	<0.0001
Number sampled by age group, n (Weighted %; 95% CI)				
< 1 years	184 (11.6; 10.3–12.9)	158 (15.3)^a^	26 (7.45; 4.69–10.2)	<0.0001
1–4 years	903 (88.4; 87.1– 89.7)	582 (84.7)^a^	321 (92.5; 89.8–95.3)	
Weighted mean number of months living in the community (95% CI)	28.1 (27.2–29.0)	26.1 (25.0–27.1)	30.4 (28.8–31.9)	<0.0001
Weighted mean number of people sleeping in the same room	4.4 (4.3–4.5)	5.1 (4.9–5.2)	3.7 (3.5–3.8)	<0.0001
Number of children under 5 years in the home, n (Weighted %; 95% CI)				
1	600 (58.3; 55.2–61.4)	379 (53.1; 49.1–57.1)	221 (64.0; 59.2–68.8)	<0.0001
2	424 (36.1; 33.0–39.1)	326 (43.3; 39.3–47.3)	98 (28.0; 23.4–32.6)	
≥ 3	63 (5.7; 4.2–7.2)	35 (3.6; 2.4–4.9)	28 (8.0; 5.2–10.8)	
Number of days per week the child attends school or daycare per week, n (Weighted %; 95% CI)				
None	618 (51.6; 48.5–54.8)	480 (62.2; 58.3–66.1)	138 (39.9, 34.8–45.1)	<0.0001
Tobacco smoke in the home (Weighted %; 95% CI)	132 (13.2; 11.0 –15.5)	71 (9.3; 7.0–11.6)	61 (17.6; 13.6–21.6)	0.0002
Current illness^b^ (Weighted %; 95% CI)				
Cough	481 (51.2; 48.5 –53.9)	391 (64.2; 61.8–66.7)	90 (33.0; 27.3–38.2)	<0.0001
Runny nose	622 (54.3; 51.2–57.4)	486 (68.0; 64.3–71.6)	136 (39.1; 34.0–44.2)	<0.0001
Fever within 24 h	146 (14.7; 12.3–17.0)	85 (12.2; 9.5–14.9)	61 (17.4; 13.5–21.3)	0.03
Recent illness (within 30 days) (Weighted %; 95% CI)				
Cough	534 (45.6; 42.4–48.7)	413 (55.2; 51.2–59.2)	121 (35.0; 30.1–40.0)	<0.0001
Pneumonia	55 (4.7; 3.3– 6.0)	47 (6.8; 4.7–8.8)	8 (2.5; 0.8–4.2)	0.007
Fast breathing	136 (12.4; 10.2–14.5)	94 (12.7; 10.0–15.3)	42 (12.0; 8.8–15.4)	0.77
Fever	464 (40.2; 37.0–43.3)	344 (45.0; 41.0–49.0)	120 (34.7; 29.7–39.7)	0.002
Any antibiotic use (Weighted %; 95% CI)				
Current^b^	59 (5.6; 4.1–7.1)	32 (3.6; 2.3–4.9)	27 (7.8; 5.0–10.6)	0.003
Within the past 7 days^b^	201 (18.9; 16.4–21.5)	123 (15.8; 12.9–18.7)	78 (22.5; 18.1–26.9)	0.01
Within the past 30 days^b^	413 (37.7; 34.5–40.8)	289 (20.8; 18.7– 22.9)	124 (16.9; 14.5–19.3)	0.19

^a^95% confidence not applicable because Kibera sample was stratified by children aged <1 years and 1 to 4 years, and therefore the variance is 0

^b^Current refers to the day of interview. The categories of antibiotic use in this table are not mutually exclusive (i.e., “within the past 30 days” include those who reported current use and use within the past 7 days)

Self-reported tobacco smoke in the home, history of recent illnesses, and antibiotic use are summarized in Table 1. Overall, more than half of children reported the presence of upper respiratory symptoms (i.e., cough, runny nose) at the time of the survey with a significantly higher proportion of children in Kibera with these symptoms compared to Lwak (64.2% vs. 33.0% with cough, 68.0% vs. 39.1% with runny nose, both *P* <0.0001). Thirty-eight percent reported antibiotic use (i.e., cotrimoxazole, penicillin/ampicillin/amoxicillin, doxycycline, chloramphenicol) within the 30 days prior to the day of the survey, and more children from Lwak reported recent antibiotic use compared to Kibera (i.e., current use or use within the past 7 days).

### Pneumococcal colonization among children

Overall, 90.0% of children were colonized with pneumococci, and 37.3 and 48.6% were colonized with PCV10 and PCV13 serotypes, respectively (Table 2). Of those with pneumococcal colonization, 4.5% (95% CI: 3.0–6.0%) had more than one serotypes identified. We found no statistically significant differences when comparing results of the 2009 and 2010 surveys in the proportion of children who were carrying pneumococcus or the proportion of isolates that were PCV10 serotypes (Additional file 3: Table S1). Therefore, results from both surveys were combined. Children aged <1 year were more likely to be colonized with pneumococci than children aged 1–4 years (95.1% vs. 89.3%, *P* = 0.009; Table 2). There were no statistically significant differences between Kibera and Lwak in the proportion of children colonized with pneumococcus, the proportion of those colonized with PCV10 or PCV13 serotypes, or pneumococcal colonization by different age groups (Table 2).

**Table 2:** Pneumococcal colonization of children in Kibera and Lwak, 2009–2010 surveys combined

Serotype of colonized pneumococcus	Total (*N* = 1,087)	Kibera (*N* = 740)	Lwak (*N* = 347)	*P* value, Kibera vs. Lwak
	n (Weighted %; 95% CI)	n (Weighted %; 95% CI)	n (Weighted %; 95% CI)	
Any serotype	983 (90.0; 88.0–92.0)	677 (91.5; 89.3–93.8)	306 (88.3; 84.9–91.6)	0.10
PCV10 type	408 (37.3; 34.2–40.5)	287 (39.4; 35.4–43.4)	121 (35.0; 30.0–40.0)	0.18
PCV13 type	532 (48.6; 45.4–51.9)	365 (49.0; 45.0–53.0)	167 (48.2; 43.0–53.5)	0.82
Colonization by age group				
< 1 year	173* (95.1; 92.3–98.0)	147 (93.0; 89.0–97.1)	26 (100)	NA^a^
1–4 years	810* (89.3; 87.1–91.5)	530 (91.3; 88.7–93.8)	280 (87.3; 83.7–90.9)	0.07

*NA* not applicable

*P = 0.009 comparing pneumococcal colonization among all children aged <1 years vs. 1–4 years

^a^ *P* value was not calculated as at least one of the cell counts were <5

### Serotype distribution of pneumococcal isolates

**Fig. 1:**
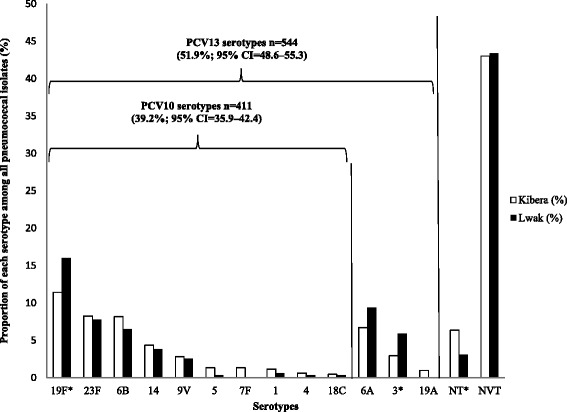
Serotype distribution of pneumococcal isolates — Kibera and Lwak, 2009–2010 surveys combined (*N* = 1,041). All % are weighted. NT: nontypeable, NVT: non-vaccine serotypes *P < 0.05

Of the 1,041 pneumococcal isolates, the most frequently isolated vaccine-type serotypes were 19F (13.5%, 95% CI 11.2–15.8), 23F (8.0%, 95% CI 6.2–9.8), 6A (7.9%, 95% CI 6.1–9.7; contained only in PCV13), and 6B (7.4%. 95% CI 5.7–9.2). Overall, 39.2% of all pneumococcal isolates were PCV10 serotypes, and 51.9% were PCV13 serotypes (Fig. 1). Serotypes 19F and 3 were more frequent among Lwak isolates compared to Kibera isolates (16.0% vs. 11.4% for serotype 19F, 5.9% vs. 2.9% for serotype 3; both *P* <0.05). Serotype 7F was only detected from Kibera isolates, and nontypeable pneumococci were isolated more frequently from the Kibera isolates (3.4% vs. 1.4%; *P* <0.05). Among the non-vaccine serotypes, 35B (3.5%), 15B (3.4%), 19B (3.4%), and 11A (3.3%) were among the most common, and nontypeable isolates constituted 4.9% of the isolates (Additional file 3: Table S2).

### Antibiotic susceptibility

Six hundred fifty-seven pneumococcal isolates collected from 624 children (90% of those enrolled in 2009) were tested for antibiotic susceptibility. Cotrimoxazole nonsusceptibility was found in 98.6% (95% CI 97.8–99.4%) of the isolates, penicillin nonsusceptibility in 81.9% (95% CI 78.7–85.2%), tetracycline nonsusceptibility in 19.2% (95% CI 15.8–22.6), and <5% nonsusceptibility for chloramphenicol (1.9% [95% CI 0.7–3.1%]), erythromycin (0.9% [95% CI 0.3–1.5%]), and clindamycin (0.1% [95% CI 0–0.3%]). All tested isolates were susceptible to levofloxacin and ceftriaxone. Penicillin nonsusceptibility was driven largely by the large proportion of isolates with intermediate susceptibility to penicillin (Table 3); MIC50’s and MIC90’s for the strains tested were both 0.5 μg/ml for penicillin-intermediate isolates and 2 and 4 μg/ml, respectively, for penicillin-resistant isolates.

**Table 3:** Number and proportion of pneumococcal isolates from children aged <5 years in Kibera and Lwak, 2009, that are susceptible, intermediate, and resistant by antibiotic (*N* = 657)

Antibiotic	Susceptible		Intermediate		Resistant	
	Break-point* μg/ml	*N* (%)	Break-point* μg/ml	*N* (%)	Break-point* μg/ml	*N* (%)
Penicillin^a^	*≤0.06*	118 (18.6)	*0.12–1*	500 (79.0)	*≥2*	15 (2.4)
Chloramphenicol^b^	*≤4*	615 (98.1)	*n/a*	n/a	*≥8*	12 (1.9)
Levofloxacin^b^	*≤2*	627 (100)	*4*	0	*≥8*	0
Erythromycin^a^	*≤0.25*	625 (98.7)	*0.5*	1 (0.2)	*≥1*	7 (1.1)
Ceftriaxone^a^	*≤1*	633 (100)	*2*	0	*≥4*	0
Tetracycline^a^	*≤2*	511 (80.7)	*4*	15 (2.4)	*≥8*	107 (16.9)
Cotrimoxazole^c^	*≤0.5/9.5*	12 (1.9)	*1/19–2/38*	58 (9.2)	*≥4/76*	561 (88.9)
Clindamycin^a^	*≤0.25*	632 (99.8)	*0.5*	0	*≥1*	1 (0.2)

*Breakpoints defined using Clinical and Laboratory Standards Institute (CLSI) guidelines 2012. For penicillin, breakpoints for oral penicillin were used

^a^24 isolates missing information on susceptibility

^b^30 isolates missing information on susceptibility

^c^26 isolates missing information on susceptibility

**Fig. 2:**
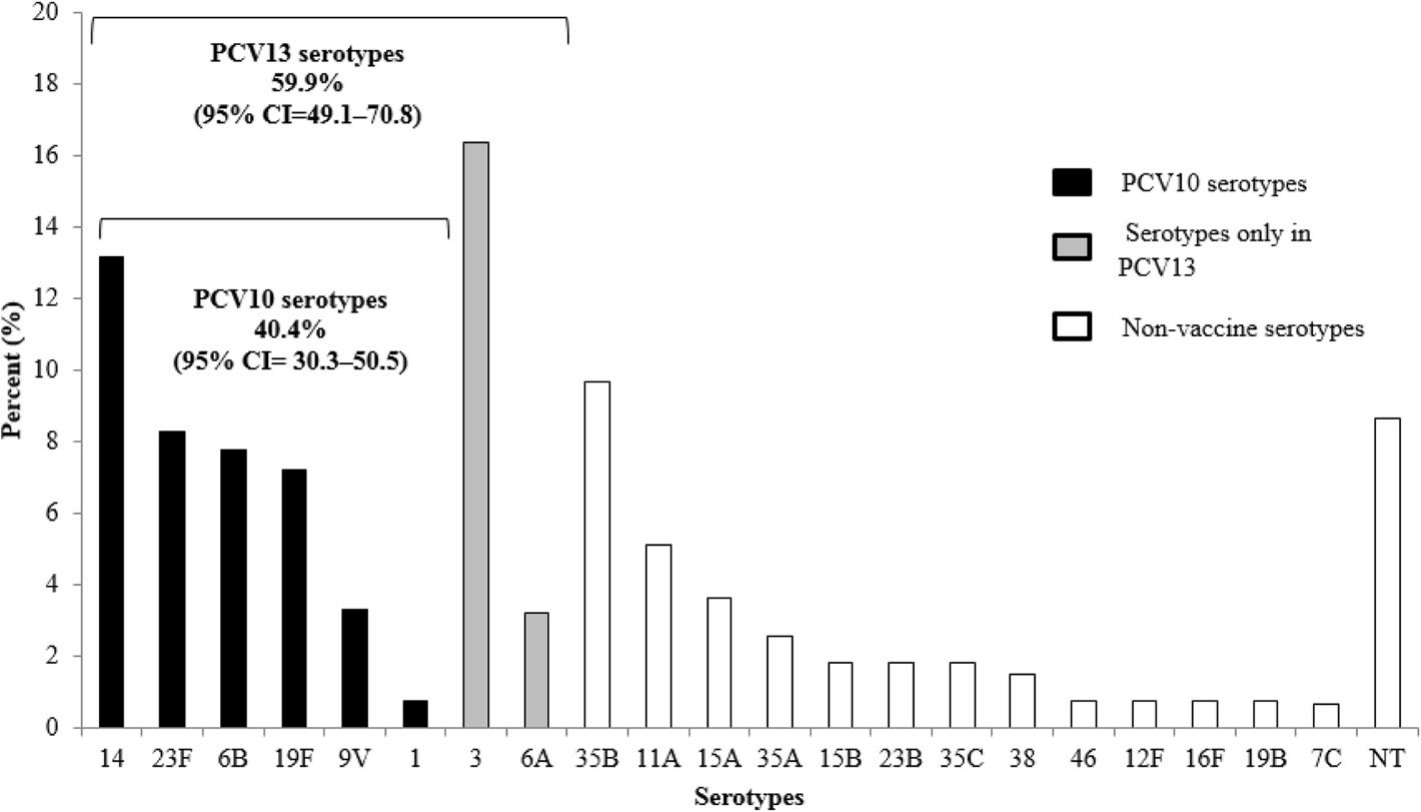
Serotype distribution of multidrug-resistant isolates — Kibera and Lwak, 2009 (*N* = 103). Multidrug-resistance was not detected in serotypes 4, 5, 7 F, 18C, and 19A. All % are weighted

MDR was found in 15.9% (95% CI 12.8–19.1%) of isolates. All MDR isolates were nonsusceptible to cotrimoxazole, and >90% of MDR isolates were nonsusceptible to both penicillin and cotrimoxazole (Additional file 3: Table S3). There were no statistically significant differences between the two sites in the proportion of MDR isolates (16.7% for Kibera vs. 15.1% for Lwak; *P* = 0.64). PCV10 serotypes and PCV13 serotypes accounted for 40.4 and 59.9% of the MDR isolates, respectively (Fig. 2).

### Factors associated with colonization with antibiotic non-susceptible pneumococci

PCV10 serotypes (PR: 1.2, 95% CI 1.1–1.3) and current amoxicillin use (PR: 1.2, 95% CI 1.1–1.4) were associated with penicillin nonsusceptibility in the multivariable model, but not recent use (i.e., within 7 days or 30 days before the survey) (Table 4). Another model showed that PCV10 serotypes were also associated with cotrimoxazole nonsusceptibility (Additional file 3: Table S4). None of the reviewed factors were statistically significantly associated with colonization with MDR pneumococcus in multivariable analysis. (Additional file 3: Table S5).

**Table 4:** Factors associated with penicillin non-susceptibility among children in Kibera and Lwak, 2009

Characteristic	Total number of children *N* = 694	PCN-nonsusceptible *N* = 490 (Weighted %; 95% CI)	Unadjusted prevalence ratio (95% CI)	Adjusted prevalence ratio (95% CI)
Place of residence				
Lwak	155	125 (80.6; 74.4–86.9)	Ref	ref
Kibera	469	365 (77.7; 73.9–81.5)	0.96 (0.88–1.06)	0.94 (0.85–1.03)
Serotype group				
NVT	368	267 (74.1; 69.3–79.0)	Ref	ref
PCV10 serotype	256	223 (86.6; 81.9–91.3)	1.17 (1.08–1.27)*	1.17 (1.07–1.27)*
Age group				
< 1 years	101	85 (85.1; 77.5–92.7)	Ref	ref
1–4 years	523	405 (78.1; 74.2–82.0)	0.92 (0.83–1.02)	0.94 (0.84–1.04)
Number of children aged <5 years in the home				
1	312	241 (76.9; 71.7–82.2)	Ref	Ref
2 or more	312	249 (81.2; 76.6–85.9)	1.06 (0.97–1.15)	1.06 (0.97–1.15)
Number of days attending school or daycare per week				
0	357	288 (81.0; 76.5–85.4)	Ref	Ref
1 or more	265	201 (77.2; 71.8–82.7)	0.95 (0.87–1.04)	0.94 (0.86–1.04)
Recent illness (within 30 days, compared to those with no illness)				
No cough	297	235 (80.7; 75.9–85.5)	Ref	Ref
Cough	327	255 (77.4; 72.3–82.5)	0.96 (0.88–1.05)	0.98 (0.89–1.08)
No pneumonia	589	460 (78.7; 75.1–82.3)	Ref	Ref
Pneumonia	35	30 (85.5; 72.4–98.6)	1.09 (0.93–1.28)	1.07 (0.91–1.27)
No fast breathing	543	424 (78.9; 69.9–89.8)	Ref	Ref
Fast breathing	81	66 (79.8; 69.9–89.8)	1.01 (0.89–1.16)	1.03 (0.89–1.19)
No fever	345	273 (80.6; 76.1–85.1)	Ref	Ref
Fever	279	217 (77.0; 71.4–82.5)	0.95 (0.87–1.05)	0.95 (0.85–1.05)
Penicillin, ampicillin, or amoxicillin use (compared to those with no use)^b^				
No current use	610	477 (78.6; 75.0–82.2)	Ref	Ref
Current use^a^	14	13 (95.3; 86.1–100)	1.21 (1.09–1.35)	1.22 (1.07–1.39)*
No use within the past 7 days	572	449 (79.1; 75.4–82.8)	Ref	Ref
Within the past 7 days	52	41 (78.3; 65.8–90.8)	0.99 (0.84–1.17)	1.00 (0.84–1.19)
No use within the past 30 days	501	393 (79.3; 75.4–83.2)	Ref	Ref
Within the past 30 days	123	97 (77.7; 69.4–85.9)	0.98 (0.87–1.10)	0.98 (0.86–1.11)

*NVT* non-vaccine type

*Statistically significant

^a^Current refers to the day of interview

^b^The categories of antibiotic use in this table are not mutually exclusive (i.e., “within the past 30 days” include those who reported current use and use within the past 7 days), therefore, the adjusted prevalence ratios “within the past 7 days” and “within the past 30 days” were calculated using separate models which only include one antibiotic use category

## Discussion

Before the introduction of PCV10 in Kenya, almost all (90%) Kenyan children aged <5 years were colonized with pneumococci, and 37.3% were colonized with PCV10 serotypes. Most isolates were not susceptible to penicillin and cotrimoxazole; PCV10 serotypes were more likely to be nonsusceptible to penicillin and cotrimoxazole than non-PCV10 serotypes, and PCV10 serotypes accounted for >40% of the MDR isolates. Notably, the high prevalence of colonization did not differ for samples collected in the urban (Kibera) and rural (Lwak) sites, in spite of differences in the frequency of certain characteristics for children enrolled in the two sites, such as the number of children aged <5 years in the household or the number of children in the household attending school or daycare.

The prevalence of pneumococcal colonization found in our study is high compared to what has been reported in previous studies. A pre-vaccine colonization study completed in Kilifi, Kenya in 2009 and 2010 found 74% pneumococcal colonization among children aged <5 years [46]. A meta-analysis of pre-PCV pneumococcal colonization studies in children aged <5 years showed an overall colonization prevalence of 64.8% (95% CI 49.8–76.1%) for low income countries (Bangladesh, the Gambia, Kenya, and Tanzania) and 47.8% (95% CI 44.7–50.8%) for lower-middle income countries (Fiji, Gaza strip, Chana, India, Indonesia, and Vietnam) [30], although one study from the Gambia [47] had similarly high prevalence (93%, 95% CI 89.9 –95.2) as our study. In addition to the crowded living conditions observed in our study population, which has been shown to intensify pneumococcal transmission [48–50], we attribute the high pneumococcal recovery rate to the broth enrichment step that was used in our study, but not in the studies included in the aforementioned meta-analysis [51–60] or the Kilifi study [46]. This specific broth enrichment combining STHB, yeast, and rabbit serum has been shown to improve pneumococcal isolation compared to conventional culture-based results by 16% [38]. Although the detected colonization prevalence was higher in our study, the proportion of PCV10-serotypes (37.3%) among children aged <5 years was similar to the results reported in other studies from Kenya, the Gambia and Nigeria [31, 46, 61, 62].

Another notable finding from our study was the large proportion of pneumococcal isolates that were nonsusceptible to penicillin (81.9%) or cotrimoxazole (98.6%). Although direct comparisons cannot be made between different studies, previous reports of pneumococcal penicillin susceptibility in Kenya suggest that there may be an increase in the proportion of penicillin nonsusceptible pneumococci over the years: a study published in 1997 reported that 60.8% of pneumococcal isolates were of intermediate susceptibility (MIC 0.12–1 μg/ml) to penicillin [63]; another study published in 2005 reported that 77% of the pneumococcal isolates had intermediate susceptibility [64]. Neither study found any penicillin resistant (MIC ≥2 μg/ml) pneumococci, whereas 2.4% of the tested isolates in our study were penicillin resistant.

Several factors have been thought to contribute to the emergence of drug-resistant pneumococcal isolates in Kenya [65]. The high burden of respiratory infections, diarrhea, HIV/AIDS and a variety of other infectious diseases may have resulted in frequent use of antibiotics. In our study, more than one-third of the participants reported being on antibiotics within the month prior to the survey. Cotrimoxazole and penicillin are two of the most commonly available antibiotics in the country, and HIV-infected individuals are more likely to be exposed to cotrimoxazole for prophylaxis [66], which has been associated with development of cotrimoxazole-resistant pneumococci [67]. Healthcare practices are also possible factors influencing emergence of drug resistance. Self-medication is quite common due to inadequate access to formal healthcare and the wide availability of antibiotics without prescriptions.

It is known that drug-resistance is more likely to be observed among pneumococcal vaccine serotypes [68–72], and PCV10 serotypes were associated with penicillin and cotrimoxazole nonsusceptibility in the multivariable model, although there was not a statistically significant association with MDR. While amoxicillin use at the time of the survey was associated with penicillin nonsusceptibility, none of the other factors, such as antibiotic use within a week or a month of the survey, or attending school or daycare [73, 74] were associated with increased risk of antibiotic resistance. There are several possibilities that may explain this observation. First, since antibiotic use and history of illness relied on self-report from the participants, it is possible that the information was not accurate due to problems with recall. Second, given that the study targeted children aged <5 years, who would not usually attend school, and since two-thirds of participants lived in crowded conditions (Kibera), most of the transmission may have occurred inside households or communities. This could have diluted the potential influence of attendance at school or daycare attendance.

We used existing data to estimate the expected reduction of PCV10 serotype colonization in our study population after vaccine introduction. Using estimated vaccine effectiveness of 60% for direct [46, 75] and 50% for indirect [75, 76] effects of PCV10 on reducing PCV10 serotype colonization among young children, an estimated PCV10 immunization coverage rate of approximately 80% in Kenya [77], and 37.3% pre-vaccine PCV10 serotype colonization prevalence from our study, we estimate that PCV10 introduction would result in a 54% reduction of PCV10-type pneumococcal colonization among children aged <5 years in Kenya early in the PCV10 program. Later, reduced transmission could reduce PCV10-serotype colonization further. Reduction in colonization does not translate directly to a reduction in IPD, and certain serotypes, such as serotypes 1 and 5, which are associated with IPD [21], are rarely isolated in colonization studies [78, 79]; in fact, surveillance results on pneumococcal disease in the region have shown that PCV10 will have provided coverage for >70% of children age <5 years with invasive pneumococcal disease [80]. Additionally, PCV introduction has been shown to reduce IPD caused by both penicillin-nonsusceptible and MDR pneumococci [72, 81]; similar effects are expected in Kenya after PCV10 introduction.

Our study has several limitations. First, since this was a cross-sectional study, the results provide only a snapshot in time of pneumococcal colonization in this population. However, our study includes data from two years from two different settings (urban and rural), and we demonstrated that the distribution of serotypes and prevalence of colonization did not differ between the two years or by study setting. Second, data collection of key variables, including history of symptoms and antibiotic use, were based on self-report and might not have been accurate due to recall. Third, since HIV status of the children were available for only a small proportion of those enrolled, association between HIV status and nonsusceptible pneumococci could not be assessed. Fourth, the relatively high proportion of antibiotic use within a month prior to the survey could have cleared the antibiotic susceptible isolates and have resulted in an overestimation of antibiotic nonsusceptible isolates. Lastly, children were sampled from surveillance records conducted in two sites in Kenya, and there might be differences between those who were under surveillance and those who were not. However, given that results from the two very different populations did not differ, our findings are likely representative of much of the Kenyan population.

## Conclusions

In conclusion, our study was able to characterize pneumococcal colonization and antibiotic susceptibility patterns among children aged <5 years in two sites in Kenya prior to PCV10 introduction. Our carriage study showed high pneumococcal colonization and prevalence of antibiotic nonsusceptibility for penicillin and cotrimoxazole, which were the main contributors to MDR. PCV10 serotypes were independently associated with penicillin and cotrimoxazole nonsusceptibility. Successful PCV10 introduction in Kenya is likely to result in substantial reductions of vaccine-type pneumococcal disease and drug-resistant pneumococcal infections, including MDR, given that PCV10 contains serotypes that are more likely to cause IPD and that 40% of colonized MDR isolates were PCV10 serotypes. Changes in the pneumococcal colonization prevalence, serotype distribution, and prevalence of antibiotic nonsusceptible isolates should be closely followed in the post-vaccine surveys. Success in Kenya could help impact vaccine policy decisions in other resource-limited settings.

## Additional files

Additional file 1: 2009 survey questionnaire. (DOC 72 kb)
Additional file 2: 2010 survey questionnaire. (DOC 81 kb)
Additional file 3:
**Table S1.** Comparison of pneumococcal colonization between the 2009 and 2010 surveys. **Table S2.** Serotype distribution of non-vaccine type pneumococci (*N* = 497). **Table S3.** Antibiotic resistance patterns of multidrug-resistant pneumococcal isolates (*N* = 103). **Table S4.** Factors associated with cotrimoxazole nonsusceptibility among children in Lwak and Kibera, 2009. **Table S5.** Factors associated with multidrug-resistance among children in Lwak and Kibera, 2009. (DOCX 40 kb)

## Abbreviations

CDC: Centers for disease control and prevention
CI: Confidence interval
CLSI: Clinical and laboratory standards institute
IEIP: International emerging infections program
IPD: Invasive pneumococcal disease
KEMRI: Kenya medical research institute
MDR: Multidrug resistant
MIC: Minimum inhibitory concentrations
NP: Nasopharyngeal
PBIDS: Population-based infectious disease surveillance
PCV: Pneumococcal conjugate vaccine
PCV10: 10-valent pneumococcal conjugate vaccine
PCV13: 13-valent pneumococcal conjugate vaccine
PR: Prevalence ratio
STHB: Supplemented Todd-Hewitt broth
