# Supplementary material for: Pneumococcal carriage and antibiotic susceptibility patterns from two cross-sectional colonization surveys among children aged <5 years prior to the introduction of 10-valent pneumococcal conjugate vaccine — Kenya, 2009–2010
Source: BMC Infect Dis. 2017 Jan 5;17:25. doi: 10.1186/s12879-016-2103-0 (PMC5217209; doi:10.1186/s12879-016-2103-0)
Supplement: Additional file 1: — 2009 survey questionnaire. (DOC 72 kb) [file 12879_2016_2103_MOESM1_ESM.doc]

**CHILD**

Place label here

or write ID Number

__________

**Pneumococcal Carriage Study Questionnaire**

| **Part I: Inclusion Criteria** | | | |
| --- | --- | --- | --- |
| 1. The participant is a resident of:  Lwak/Asembo DSS area  Kibera DSS area | | | |
| 2. Has the participant’s primary residence been in either of these communities for at least four months? |  Yes |  No |  Declined |
| 3. Is the participant:  a child aged < 5 years  the parent of a child aged < 5 years | | | |
| 1. Does the participant have a voucher indicating their selection to participate in this study? |  Yes |  No |  Declined |

***If the participant does NOT meet ALL of the above criteria, STOP here and thank them for their time.***

***If the participant meet ALL of the above criteria, please complete the appropriate consent form and proceed with the interview.***

***Use the Parent questionnaire for adult participants***

***and***

***Use the Child questionnaire for children ages 0-5 years***

| **Part II: Participant Demographics** | | |
| --- | --- | --- |
| First Name: | Juok (Middle) Name: | Last (Father’s) Name: |
| DDS Permanent ID number: | | |

***Upon completion of the questionnaire, please separate this page from the following page and store in separate location from rest of data.***

Place label here

or write ID Number

__________

**Pneumococcal Carriage Study Questionnaire – CHILD**

| **Part III: General information** | |
| --- | --- |
| Date of Interview (dd/mm/yyyy) : ________/__________/__________ | Interviewer Code: ____________________ |
| Consent obtained  Yes  No  Don’t know (if *No* or *Don’t know*, stop questionnaire and obtain informed consent) | |

| **Part IV: Participant Information** | | | | | |
| --- | --- | --- | --- | --- | --- |
| 1. Age: _____ years (if less than 1 year: ________ months) | | | 2. Gender:  Male  Female | | |
| 3. How long has the child lived in this community (Lwak/Asembo)? __________ years  (if less than 1 year: ________ months) | | | | | |
| 4. How many people sleep in the same room as the child (total, including participant) _____________ | | | | | |
| 5. How many children living in the same household as the child attend primary school or daycare?  (total, including participant) _______________ | | | | | |
| 6. How many days per week does the child attend school or daycare? (circle the number of days)  0 1 2 3 4 5 6 7 | | | | | |
| 7. **Please list the current age of each child’s < 5 years old living in the same household as the participant**:  PLEASE LIST THE PARTICIPANT FIRST (use other side if needed) | | | | | |
|  | Number of PCV vaccinations |  | | | Number of PCV vaccinations |
| a. PARTICIPANT |  0  1  2  3  unknown | e. Age: _____ | | |  0  1  2  3 unknown |
| b. Age: ______ |  0  1  2  3  unknown | f. Age: ______ | | |  0  1  2  3  unknown |
| c. Age: ______ |  0  1  2  3  unknown | g. Age: _____ | | |  0  1  2  3 unknown |
| d. Age: ______ |  0  1  2  3  unknown | h. Age: _____ | | |  0  1  2  3 unknown |
| 8. **In the previous month,** what type of fuel has your household usually used for cooking?  (select all that apply): | | | | - Firewood / wood - Crop waste - Charcoal - Kerosene or paraffin - Dung - Electricity - Sawdust - Other (describe) _________________ | |

| 9. **In the previous month, what kind of heat source has your household usually used for cooking?**  (select one) | | | - Fire pit - Paraffin stove - Jiko stove - Rocket stove (block stove) - Electrical or gas cooker - Other (describe) ________________________________ | | | |
| --- | --- | --- | --- | --- | --- | --- |
| 10. In the previous month, where has the cooking usually been done?  (select one): | | - A separate building dedicated for cooking (such as a cooking hut) - The same area where you live or sleep (such as a single hut with a cooking pit) - The house where you live, but in a separate room used as a kitchen (a kitchen with walls) - Outdoors / outside the house (for example, just outside the hut wall) - Other ______________________________ | | | | |
| 11. In the previous month, have there been any other times besides cooking that your child was exposed to smoke inside or outside of the house? (please read options to participant and select all that apply):   - Yes, when we heat the house with a fire - Yes, when we keep a fire burning to keep away mosquitoes - Yes, after cooking as the embers burn out - Yes, when we use fire for light - Yes, when we use tin lamps - Yes, we keep a fire burning for other reasons (describe) ______________ - No | | | | | | |
| 12. Does the child currently have a cough? | | | | |  yes  no  don’t know  refused | |
| 13. **Has the child had a cough within the past 30 days?** | | | | |  yes  no  don’t know  refused | |
| 14. Does the child currently have a runny nose (coryza)? | | | | |  yes  no  don’t know  refused | |
| 15. Has the child had a fever in the last 24 hours? | | | | |  yes  no  don’t know  refused | |
| **16. Has the child had a fever within the past 30 days?** | | | | |  yes  no  don’t know  refused | |
| **17. Has the child had any fast breathing within the past 30 days?** | | | | |  yes  no  don’t know  refused | |
| **18. Has the child had pneumonia within the past 30 days?** | | | | |  yes  no  don’t know  refused | |
| 19. If you don’t know your child’s HIV status, would you like them to be tested today? | | | | |  yes  no  don’t know  refused | |
| 20. Does anyone in your home smoke tobacco? | | | | |  yes  no  don’t know  refused | |
| 21. We would like to know if your child has taken any antibiotics recently. Has the child taken any antibiotics. | | | | | | |
|  | Today? | | | Within the past 7 days? | | Within the past 30 days? |
| septrin/  cotrimoxazole |  yes  no  don’t know | | |  yes  no  don’t know | |  yes  no  don’t know |
| amoxicillin/ ampicillin/penicillin |  yes  no  don’t know | | |  yes  no  don’t know | |  yes  no  don’t know |
| doxycycline or  tetracycline |  yes  no  don’t know | | |  yes  no  don’t know | |  yes  no  don’t know |
| chloramphenicol |  yes  no  don’t know | | |  yes  no  don’t know | |  yes  no  don’t know |
| any other antibiotic1 *(list)*_____________  _________________ |  yes  no  don’t know | | |  yes  no  don’t know | |  yes  no  don’t know |
| any other antibiotic 2 *(list)*_____________  _________________ |  yes  no  don’t know | | |  yes  no  don’t know | |  yes  no  don’t know |

*Thank the participant for their time and proceed to sample collection.*
