# Supplementary material for: Pneumococcal carriage and antibiotic susceptibility patterns from two cross-sectional colonization surveys among children aged <5 years prior to the introduction of 10-valent pneumococcal conjugate vaccine — Kenya, 2009–2010
Source: BMC Infect Dis. 2017 Jan 5;17:25. doi: 10.1186/s12879-016-2103-0 (PMC5217209; doi:10.1186/s12879-016-2103-0)
Supplement: Additional file 2: — 2010 survey questionnaire. (DOC 81 kb) [file 12879_2016_2103_MOESM2_ESM.doc]

Place label here

or write ID Number

__________

**Pneumococcal Carriage Study Questionnaire**

| **Part I: Inclusion Criteria** | | | |
| --- | --- | --- | --- |
| 1. The participant is a resident of:  Lwak  Kibera | | | |
| 2. Has the participant’s primary residence been in either of these communities for at least four months? |  Yes |  No |  Declined |
| 3. Is the participant:   a child aged < 5 years | | | |
| 4. Does the participant have a card indicating their selection to participate in this study? |  Yes |  No |  Declined |

***If the participant does NOT meet ALL of the above criteria, STOP here and thank them for their time.***

***If the participant meets ALL of the above criteria, please complete the appropriate consent form and proceed with the interview.***

| **Part II: Participant Demographics** | | | | |
| --- | --- | --- | --- | --- |
| First Name*:* | Middle Name*:* | | Last (Father’s) Name: | |
| Permanent ID number: --- | | | | |
| **Parent or Guardian Demographics** (Please fill in the following information about the adult who accompanied the participant) | | | | |
| Parent/Guardian First Name: | | Parent/Guardian Middle Name | | Parent/Guardian Last (Father’s) Name |
| Relationship to child: | | | | |

***Upon entering the questionnaire in the database, please separate this page from the following page and store in separate location from rest of data.***

***DO NOT USE OR DELETE THIS PAGE***

**Pneumococcal Carriage Study Questionnaire: English**

| **Part III: General information** | |
| --- | --- |
| Date of Interview (dd/mm/yyyy) :  // | Interviewer Code:   |
| Consent obtained?  Yes  No Don’t know  (if *No* or *Don’t know*, stop questionnaire and obtain informed consent) | |

| **Part IV: Participant Information** | | | | |
| --- | --- | --- | --- | --- |
| 1. Age:  years  (if less than 1 year:  months) | | | 2. Gender:   Male  Female | |
| 3. How long has the child lived in this community?   years (if less than 1 year:  months) | | | | |
| 4. How many people sleep in the same room as the child (total, including participant)?  people | | | | |
| 5. How many children living in the same household as the child attend primary school or daycare?  (total, including participant) children | | | | |
| 6. How many days per week does the child attend school or daycare? (circle the number of days)  0 1 2 3 4 5 6 7 | | | | |
| 7. Please list the age and PCV vaccination status of each child <5 years old living in the home as indicated on their vaccination cards*:*  PLEASE LIST THE PARTICIPANT FIRST | | | | |
|  | Number of PCV vaccinations |  | | Number of PCV vaccinations |
| a. PARTICIPANT |  0  1  2  3 unknown | e. Age:  ___years __months | |  0  1  2  3 unknown |
| b. Age:  ___years __months |  0  1  2  3 unknown | f. Age:  ___years __months | |  0  1  2  3 unknown |
| c. Age:  ___years  __months |  0  1  2  3 unknown | g. Age:  ___years  __months | |  0  1  2  3 unknown |
| d. Age:  ___years __months |  0  1  2  3 unknown | h. Age:  ___years __months | |  0  1  2  3 unknown |

| **8. Please list the number of doses of influenza vaccinations this child has received in the past year as indicated on their vaccination cards:** | | | | | | ** 0  1  2  3+ unknown** | |
| --- | --- | --- | --- | --- | --- | --- | --- |
| 9. **In the previous month,** what type of fuel has your household usually used for cooking?  **(select all that apply):** | | | | | | - Firewood - Crop waste - Charcoal - Kerosene or paraffin - Dung - Electricity - Sawdust - Other (describe) ___________________ | |
| 10. **In the previous month,** what kind of heat source has your household usually used for cooking?  **(select all that apply):** | | | - Fire pit - Paraffin stove - Jiko stove (charcoal) - Rocket stove - Electrical or gas cooker - Other (describe)______________________ | | | | |
| 11. **In the previous month,** where has the cooking usually been done?  **(select one):** | | - A separate building dedicated for cooking (such as a cooking hut) - The same area where you live or sleep (such as a single hut with a cooking pit) - The house where you live, but in a separate room used as a kitchen (a kitchen with walls) - Outdoors / outside the house (for example, just outside the hut wall) - Other ______________________________ | | | | | |
| Continued on next page…  12. **In the previous month,** have there been any other times besides cooking that your child was exposed to smoke inside or outside of the house? (please read all options to participant*)* Check all that apply.   - Yes, when we heat the house with a fire - Yes, we keep a fire burning to keep away mosquitoes - Yes, after cooking as the embers burn out - Yes, when we use fire for light - Yes, when we use tin lamps - Yes, we keep a fire burning for other reasons (describe) ______________ - No | | | | | | | |
| 13. Does the child currently have a cough? | | | | |  yes  no  don’t know  refused | | |
| 14. Has the child had a cough within the past 30 days? | | | | |  yes  no  don’t know  refused | | |
| 15. Does the child currently have a runny nose? | | | | |  yes  no  don’t know  refused | | |
| 16. Has the child had a fever in the last 24 hours? | | | | |  yes  no  don’t know  refused | | |
| 17. Has the child had a fever within the past 30 days? | | | | |  yes  no  don’t know  refused | | |
| 18. Has the child had any fast breathing within the past 30 days? | | | | |  yes  no  don’t know  refused | | |
| 19. Has the child had pneumonia within the past 30 days? | | | | |  yes  no  don’t know  refused | | |
| 20. If you don’t know your child’s HIV status, would you like them to be tested today? | | | | |  yes  no  don’t know  refused | | |
| 21. Does anyone in your home smoke tobacco? | | | | |  yes  no  don’t know  refused | | |
| Continued on next page ….  22. We would like to know if your child has taken any antibiotics recently. Has the child taken any antibiotics…? | | | | | | | |
|  | Today? | | | Within the past 7 days? | | | Within the past 30 days? |
| septrin/  cotrimoxazole |  yes  no   don’t know | | |  yes  no   don’t know | | |  yes  no   don’t know |
| amoxicillin/ampicillin/ penicillin |  yes  no   don’t know | | |  yes  no   don’t know | | |  yes  no   don’t know |
| doxycycline or  tetracycline |  yes  no   don’t know | | |  yes  no   don’t know | | |  yes  no   don’t know |
| chloramphenicol |  yes  no   don’t know | | |  yes  no   don’t know | | |  yes  no   don’t know |
| any other antibiotic1 *(list)*_____________  _________________ |  yes  no   don’t know | | |  yes  no   don’t know | | |  yes  no   don’t know |
| any other antibiotic 2 *(list)*_____________  _________________ |  yes  no   don’t know | | |  yes  no   don’t know | | |  yes  no   don’t know |

*End of interview*

*.*

*Thank the participant for their time and proceed to sample collection.*
