# Supplementary material for: Pneumococcal carriage and antibiotic susceptibility patterns from two cross-sectional colonization surveys among children aged <5 years prior to the introduction of 10-valent pneumococcal conjugate vaccine — Kenya, 2009–2010
Source: BMC Infect Dis. 2017 Jan 5;17:25. doi: 10.1186/s12879-016-2103-0 (PMC5217209; doi:10.1186/s12879-016-2103-0)
Supplement: Additional file 3: — Table S1. Comparison of pneumococcal colonization between the 2009 and 2010 surveys. Table S2. Serotype distribution of non-vaccine type pneumococci (N = 497). Table S3. Antibiotic resistance patterns of multidrug-resistant pneumococcal isolates (N = 103). Table S4. Factors associated with cotrimoxazole nonsusceptibility among children in Lwak and Kibera, 2009. Table S5. Factors associated with multidrug-resistance among children in Lwak and Kibera, 2009. (DOCX 40 kb) [file 12879_2016_2103_MOESM3_ESM.docx]

Additional file 1

Table S1: Comparison of pneumococcal colonization between the 2009 and 2010 surveys

|  | **Total** | **2009** | **2010** | ***P* value, 2009 vs. 2010** |
| --- | --- | --- | --- | --- |
|  | **(N=1,087)** | **(N=697)** | **(N=390)** |  |
| **Serotype of colonized pneumococcus** | n (Weighted %; 95% CI) |  | n (Weighted %; 95% CI) |  |
|  |  | n (Weighted %; 95% CI) |  |  |
| Any serotype | 983  (90.0; 88.0–92.0) | 624  (88.1; 85.3–90.9) | 359  (92.0; 89.2–94.8) | 0.06 |
| PCV10 type | 408  (37.3; 34.2–40.5) | 256  (34.7; 30.8–38.6) | 152  (40.1; 35.0–45.1) | 0.09 |

Table S2: Serotype distribution of non-vaccine type pneumococci (N=497)

| **Serotype** | **N (Weighted %; 95% CI)** |
| --- | --- |
| 19B | 35 (3.4; 2.2–4.6) |
| 35B | 34 (3.5; 2.2–4.7) |
| 11A | 34 (3.3; 2.1–4.5) |
| 15B | 33 (3.4; 2.1–4.6) |
| 15A | 29 (3.1; 1.9–4.3) |
| 13 | 26 (2.4; 1.4–3.4) |
| 34 | 23 (2.7; 1.6–3.9) |
| 15C | 22 (2.3; 1.3–3.3) |
| 16F | 21 (2.1; 1.1–3.1) |
| 7C | 20 (2.3; 1.2–3.3) |
| 23B | 18 (1.7; 0.8–2.5) |
| 10A | 18 (1.8; 0.9–2.7) |
| 20 | 15 (1.0; 0.5–1.6) |
| 18A | 13 (1.1; 0.4–1.8) |
| 10F | 12 (0.8; 0.3–1.4) |
| 23A | 9 (0.8; 0.2–1.3) |
| 17F | 9 (0.8; 0.2–1.3) |
| 38 | 8 (0.7; 0.2–1.2) |
| 6C | 7 (0.6; 0.1–1.0) |
| 12F | 7 (0.6; 0.1–1.0) |
| 35A | 6 (0.5; 0.06–0.1) |
| 33D | 4 (0.6; 0.01–1.1) |
| 21 | 4 (0.5; 0–1.0) |
| 35F | 4 (0.3; 0–0.7) |
| 46 | 4 (0.2; 0.005–0.4) |
| 24F | 4 (0.6; 0.01–1.1) |
| 9N | 2 (0.2; 0–0.5) |
| 25A | 2 (0.1; 0–0.3) |
| 9L | 2 (0.1; 0–0.3) |
| 22F | 2 (0.2; 0–0.5) |
| 33F | 2 (0.2; 0–0.5) |
| 31 | 2 (0.2; 0–0.5) |
| 35C | 1 (0.1; 0–0.4) |
| 9A | 1 (0.1; 0–0.4) |
| 39 | 1 (0.06; 0–0.2) |
| 33B | 1 (0.06; 0–0.2) |
| 10B | 1 (0.06; 0–0.2) |
| 25F | 1 (0.06; 0–0.2) |
| 5B | 1 (0.1; 0–0.4) |
| 28F | 1 (0.1; 0–0.4) |
| 22A | 1 (0.1; 0–0.4) |
| 7A | 1 (0.1; 0–0.4) |
| 18B | 1 (0.1; 0–0.4) |
| NT | 55 (4.9; 3.5–6.3) |

NT: nontypable

Table S3: Antibiotic resistance patterns of multidrug-resistant pneumococcal isolates (N=103)

| **Antibiotic*** | **Number of nonsusceptible isolates (weighted%; 95% CI)** |
| --- | --- |
| Cotrimoxazole | 103 (100) |
| Tetracycline | 100 (96.8; 92.8–100) |
| Penicillin | 98 (95.3; 90.8–99.7) |
| Chloramphenicol | 12 (12.2; 5.0–19.4) |
| Erythromycin | 8 (5.7; 1.8–9.5) |
| Clindamycin | 1 (0.7; 0–2.2) |

*None of the tested isolates were nonsusceptible to ceftriaxone and levofloxacin

Table S4. Factors associated with cotrimoxazole nonsusceptibility among children in Lwak and Kibera, 2009

| **Characteristic** | | **Total number of children**  **N=694** | **Cotrimoxazole-nonsusceptible**  **N=591**  (Weighted%; 95% CI) | **Unadjusted Prevalence Ratio**  (95% CI) | **Adjusted Prevalence Ratio**  (95% CI) |
| --- | --- | --- | --- | --- | --- |
|  | **Place of residence** | | | | |
| Lwak | | 155 | 149  (96.1; 93.1–99.2) | ref | ref |
| Kibera | | 469 | 442  (94.2; 92.0–96.3) | 0.98 (0.9 –1.02) | 0.96  (0.91–0.997)* |
|  | **Serotype group** | | | | |
| NVT | | 368 | 342  (93.5; 90.8–96.2) | ref | ref |
| PCV10 serotype | | 256 | 249  (97.5; 95.4–99.5) | 1.04 (1.01–1.08)* | 1.05  (1.01–1.08)* |
|  | **Age group** | | | | |
| <1 years | | 101 | 97  (94.2; 88.1–100) | ref | Ref |
| 1–4 years | | 523 | 494  (95.2; 93.3–97.1) | 1.01 (0.99–1.08) | 1.02  (0.95–1.09) |
|  | **Number of children aged >5 years in the home** | | | | |
| 1 | | 312 | 298  (96.0; 93.8–98.3) | ref | ref |
| 2 or more | | 312 | 293  (94.1; 91.2–96.9) | 0.98 (0.94–1.02) | 0.99  (0.96–1.03) |
|  | **Number of days attending school or daycare per week** | | | | |
| 0 | | 357 | 346  (97.1; 95.2–98.9) | ref | ref |
| 1 or more | | 265 | 243  (93.0; 89.8–96.1) | 0.96 (0.92–0.996)* | 0.95  (0.91–0.99)* |
|  | **Recent illness (within 30 days, compared to those with no illness)** | | | | |
| No cough | | 297 | 279  (95.0; 92.5 –97.4) | Ref | ref |
| Cough | | 327 | 312  (95.1; 92.5–97.8) | 1.00 (0.96–1.04) | 1.01  (0.98–1.05) |
| No pneumonia | | 589 | 558 (95.2; 93.4–97.0) | Ref | ref |
| Pneumonia | | 35 | 33  (92.2; 80.8–100) | 0.96 (0.85–1.10) | 0.97  (0.87–1.08) |
| No fast breathing | | 543 | 514  (95.0; 80.8–100) | Ref | ref |
| Fast breathing | | 81 | 77  (95.4; 90.5–100) | 1.00 (0.95–1.03) | 1.01  (0.96–1.07) |
| No fever | | 345 | 327  (95.4; 93.1–97.7) | Ref | ref |
| Fever | | 279 | 264  (94.7; 91.7–97.6) | 0.99 (0.95–1.03) | 0.99  (0.95 –1.03) |
|  | **Cotrimoxazole use (compared to those with no use)^b^** | | | | |
| No current use | | 605 | 574  (95.3; 93.5–97.1) | Ref | ref |
| Current use^a^ | | 19 | 17  (88.7; 72.6–100) | 0.93 (0.78–1.12) | 0.94  (0.79–1.11) |
| No use within the past 7 days | | 561 | 531  (95.2; 93.3–97.0) | Ref | ref |
| Within the past 7 days | | 63 | 60  (94.3; 87.6–100) | 0.99 (0.92–1.07) | 0.99  (0.92–1.06) |
| No use within the past 30 days | | 503 | 476  (95.1; 93.1–97.1) | Ref | ref |
| Within the past 30 days | | 121 | 115  (95.1; 90.8–99.3) | 1.00 (0.95–1.05) | 1.00  (0.95–1.04) |

NVT=non-vaccine type

*Chi-Square P<0.05

^a^ Current refers to the day of interview

^b^ The categories of antibiotic use in this table are not mutually exclusive (i.e., “within the past 30 days” include those who reported current use and use within the past 7 days), therefore, the adjusted prevalence ratios “within the past 7 days” and “within the past 30 days” were calculated using separate models which only include one antibiotic use category.

Table S5. Factors associated with multidrug-resistance among children in Lwak and Kibera, 2009

| **Characteristic** | | **Total number of children**  **N=694** | **Multidrug-resistant**  **N=94**  (Weighted %;  95% CI) | **Unadjusted Prevalence Ratio**  (95% CI) | **Adjusted Prevalence Ratio**  **(95% CI)** |
| --- | --- | --- | --- | --- | --- |
|  | **Place of residence** | | | | |
| Lwak | | 155 | 20 (12.9; 7.6–18.2) | Ref | ref |
| Kibera | | 469 | 74 (15.8; 12.4–19.1) | 1.22 (0.77–1.94) | 1.19 (0.75–1.89) |
|  | **Serotype group** | | | | |
| NVT | | 368 | 45 (13.0; 9.1–17.0) | Ref | Ref |
| PCV10 serotype | | 256 | 49 (16.6; 12.0–21.3) | 1.28 (0.85–1.92) | 1.28 (0.85–1.92) |
|  | **Age group** | | | | |
| <1 years | | 101 | 14 (10.6; 5.2–16.0) | ref | Ref |
| 1–4 years | | 523 | 80 (15.1; 11.7–18.5) | 1.42 (0.81–2.48) | 1.53 (0.91–2.59) |
|  | **Number of children <5 years in home** | | | | |
| 1 | | 312 | 49  (14.9; 10.6–19.2) | ref | Ref |
| 2 or more | | 312 | 45 (14.0; 9.7–18.2) | 0.93 (0.62–1.43) | 0.99 (0.65–1.50) |
|  | **Number of days attending school or daycare per week** | | | | |
| 0 | | 357 | 58  (15.3; 11.4–19.3) | Ref | Ref |
| 1 or more | | 265 | 36 (13.6; 9.1–18.2) | 0.89 (0.58–1.36) | 0.92 (0.60–1.41) |
|  | **Recent illness (within 30 days, compared to those with no illness)** | | | | |
| No cough | | 297 | 45 (14.9; 10.4–19.4) | Ref | Ref |
| Cough | | 327 | 49 (14.0; 10.0–18.1) | 0.94 (0.62–1.43) | 0.86 (0.54–1.36) |
| No pneumonia | | 589 | 91 (14.9; 11.7–18.0) | ref | Ref |
| Pneumonia | | 35 | 3 (6.9; 0–14.7) | 0.47 (0.15–1.45) | 0.41 (0.14–1.24) |
| No fast breathing | | 543 | 81 (14.3; 11.1–17.5) | Ref | Ref |
| Fast breathing | | 81 | 13 (15.7; 6.9–24.4) | 1.09 (0.60–2.00) | 1.12 (0.61–2.06) |
| No fever | | 345 | 46  (13.2; 9.2–17.1) | ref | Ref |
| Fever | | 279 | 48 (16.1; 11.4–20.8) | 1.22 (0.81–1.85) | 1.40 (0.91–2.16) |
|  | **Any antibiotic use (compared to those with no antibiotic use)^b^** | | | | |
| No current use | | 593 | 92 9  (15.1; 11.9–18.2) | ref | Ref |
| Current^a^ | | 31 | 2  (4.3; 0–10.1) | 0.28 (0.07–1.14) | 0.31 (0.08–1.23) |
| No use within the past 7 days | | 515 | 79  (14.6; 11.3–17.9) | Ref |  |
| Within the past 7 days | | 109 | 15  (13.9; 6.6–21.2) | 0.96 (0.54–1.69) | 0.98 (0.54–1.75) |
| No use within the past 30 days | | 403 | 56 (13.7; 9.9–17.4) | Ref |  |
| Within the past 30 days | | 221 | 38  (15.9; 10.7–21.1) | 1.16 (0.76–1.78) | 1.21 (0.76–1.91) |

NVT: non-vaccine type

^a^ Current refers to the day of interview.

^b^ The categories of antibiotic use in this table are not mutually exclusive (i.e., “within the past 30 days” include those who reported current use and use within the past 7 days), therefore, the adjusted prevalence ratios “within the past 7 days” and “within the past 30 days” were calculated using separate models which only include one antibiotic use category.
